# Predictive value of 8-year blood pressure measures in intracerebral haemorrhage risk over 5 years

**DOI:** 10.1093/eurjpc/zwae147

**Published:** 2024-04-17

**Authors:** Yiqian Zhang, Yinqi Ding, Canqing Yu, Dianjianyi Sun, Pei Pei, Huaidong Du, Ling Yang, Yiping Chen, Dan Schmidt, Daniel Avery, Jianwei Chen, Junshi Chen, Zhengming Chen, Liming Li, Jun Lv, China Kadoorie Biobank Collaborative Group, Junshi Chen, Zhengming Chen, Robert Clarke, Rory Collins, Liming Li, Jun Lv, Richard Peto, Robin Walters, Daniel Avery, Maxim Barnard, Derrick Bennett, Lazaros Belbasis, Ruth Boxall, Ka Hung Chan, Yiping Chen, Zhengming Chen, Charlotte Clarke, Johnathan Clarke, Robert Clarke, Huaidong Du, Ahmed Edris Mohamed, Hannah Fry, Simon Gilbert, Pek Kei Im, Andri Iona, Maria Kakkoura, Christiana Kartsonaki, Hubert Lam, Kuang Lin, James Liu, Mohsen Mazidi, Iona Millwood, Sam Morris, Qunhua Nie, Alfred Pozarickij, Maryanm Rahmati, Paul Ryder, Saredo Said, Dan Schmidt, Becky Stevens, Iain Turnbull, Robin Walters, Baihan Wang, Lin Wang, Neil Wright, Ling Yang, Xiaoming Yang, Pang Yao, Xiao Han, Can Hou, Qingmei Xia, Chao Liu, Jun Lv, Pei Pei, Dianjianyi Sun, Canqing Yu, Lang Pan, Zengchang Pang, Ruqin Gao, Shanpeng Li, Haiping Duan, Shaojie Wang, Yongmei Liu, Ranran Du, Yajing Zang, Liang Cheng, Xiaocao Tian, Hua Zhang, Yaoming Zhai, Feng Ning, Xiaohui Sun, Feifei Li, Silu Lv, Junzheng Wang, Wei Hou, Wei Sun, Shichun Yan, Xiaoming Cui, Chi Wang, Zhenyuan Wu, Yanjie Li, Quan Kang, Huiming Luo, Tingting Ou, Xiangyang Zheng, Zhendong Guo, Shukuan Wu, Yilei Li, Huimei Li, Ming Wu, Yonglin Zhou, Jinyi Zhou, Ran Tao, Jie Yang, Jian Su, Fang Liu, Jun Zhang, Yihe Hu, Yan Lu, Liangcai Ma, Aiyu Tang, Shuo Zhang, Jianrong Jin, Jingchao Liu, Mei Lin, Zhenzhen Lu, Lifang Zhou, Changping Xie, Jian Lan, Tingping Zhu, Yun Liu, Liuping Wei, Liyuan Zhou, Ningyu Chen, Yulu Qin, Sisi Wang, Xianping Wu, Ningmei Zhang, Xiaofang Chen, Xiaoyu Chang, Mingqiang Yuan, Xia Wu, Xiaofang Chen, Wei Jiang, Jiaqiu Liu, Qiang Sun, Faqing Chen, Xiaolan Ren, Caixia Dong, Hui Zhang, Enke Mao, Xiaoping Wang, Tao Wang, Xi zhang, Kai Kang, Shixian Feng, Huizi Tian, Lei Fan, XiaoLin Li, Huarong Sun, Pan He, Xukui Zhang, Min Yu, Ruying Hu, Hao Wang, Xiaoyi Zhang, Yuan Cao, Kaixu Xie, Lingli Chen, Dun Shen, Xiaojun Li, Donghui Jin, Li Yin, Huilin Liu, Zhongxi Fu, Xin Xu, Hao Zhang, Jianwei Chen, Yuan Peng, Libo Zhang, Chan Qu, Shuya Li, Haiqiang Qin, Yongjun Wang, Qiling Chen, Jihua Wang, Xiaojia Sun, Lei Wang, Xun Wang, Liming Zhang, Shanshan Zhou, Hongyuan Chen, Li Chen, Haiyan Gou, Weizhi Wang, Yanmei Zhu, Yulan Zhu, Ning Zhang, Xin Cheng, Qiang Dong, Yi Dong, Kun Fang, Yiting Mao, Yu An, Peiling Chen, Yinghua Chen, Zhihong Liu, Xiaohong Chen, Naixin Jv, Xiaojiu Li, Liyang Liu, Yun Lu, Xiaona Xing, Shihao You, Xiaoli Cheng, Chaojun Gua, Jinping Jiang, Jingyi Liu, Shumei Ma, Xuefeng Yang, Xiaomo Du, Jian Xu, Xuecheng Yang, Xiaodi Zhao, Zilong Hao, Ming Liu, Deren Wang, Xiaoting Li, Lili Hui, Zhanling Liao, Feng Liu, Chunning Feng, Dejiang Ji, Fengxia Qu, Wenwen Yuan, Jing Ding, Peng Du, Lirong Jin, Yueshi Mao, Xin Wang

**Affiliations:** Department of Epidemiology and Biostatistics, School of Public Health, Peking University, 38 Xueyuan Road, Haidian District, Beijing 100191, China; Department of Epidemiology and Biostatistics, School of Public Health, Peking University, 38 Xueyuan Road, Haidian District, Beijing 100191, China; Department of Epidemiology and Biostatistics, School of Public Health, Peking University, 38 Xueyuan Road, Haidian District, Beijing 100191, China; Peking University Center for Public Health and Epidemic Preparedness and Response, 38 Xueyuan Road, Haidian District, Beijing 100191, China; Key Laboratory of Epidemiology of Major Diseases (Peking University), Ministry of Education, 38 Xueyuan Road, Haidian District, Beijing 100191, China; Department of Epidemiology and Biostatistics, School of Public Health, Peking University, 38 Xueyuan Road, Haidian District, Beijing 100191, China; Peking University Center for Public Health and Epidemic Preparedness and Response, 38 Xueyuan Road, Haidian District, Beijing 100191, China; Key Laboratory of Epidemiology of Major Diseases (Peking University), Ministry of Education, 38 Xueyuan Road, Haidian District, Beijing 100191, China; Peking University Center for Public Health and Epidemic Preparedness and Response, 38 Xueyuan Road, Haidian District, Beijing 100191, China; Medical Research Council Population Health Research Unit at the University of Oxford, Oxford, UK; Clinical Trial Service Unit and Epidemiological Studies Unit (CTSU), Nuffield Department of Population Health, University of Oxford, UK; Medical Research Council Population Health Research Unit at the University of Oxford, Oxford, UK; Clinical Trial Service Unit and Epidemiological Studies Unit (CTSU), Nuffield Department of Population Health, University of Oxford, UK; Medical Research Council Population Health Research Unit at the University of Oxford, Oxford, UK; Clinical Trial Service Unit and Epidemiological Studies Unit (CTSU), Nuffield Department of Population Health, University of Oxford, UK; Clinical Trial Service Unit and Epidemiological Studies Unit (CTSU), Nuffield Department of Population Health, University of Oxford, UK; Clinical Trial Service Unit and Epidemiological Studies Unit (CTSU), Nuffield Department of Population Health, University of Oxford, UK; Liuyang Centers for Disease Control and Prevention, NO.11 Section 2 Lihua Road, Jili Subdistrict, Liuyang, Changsha, Hunan 410300, China; China National Center for Food Safety Risk Assessment, 37 Guangqu Road, Chaoyang District, Beijing 100022, China; Clinical Trial Service Unit and Epidemiological Studies Unit (CTSU), Nuffield Department of Population Health, University of Oxford, UK; Department of Epidemiology and Biostatistics, School of Public Health, Peking University, 38 Xueyuan Road, Haidian District, Beijing 100191, China; Peking University Center for Public Health and Epidemic Preparedness and Response, 38 Xueyuan Road, Haidian District, Beijing 100191, China; Key Laboratory of Epidemiology of Major Diseases (Peking University), Ministry of Education, 38 Xueyuan Road, Haidian District, Beijing 100191, China; Department of Epidemiology and Biostatistics, School of Public Health, Peking University, 38 Xueyuan Road, Haidian District, Beijing 100191, China; Peking University Center for Public Health and Epidemic Preparedness and Response, 38 Xueyuan Road, Haidian District, Beijing 100191, China; Key Laboratory of Epidemiology of Major Diseases (Peking University), Ministry of Education, 38 Xueyuan Road, Haidian District, Beijing 100191, China; State Key Laboratory of Vascular Homeostasis and Remodeling, Peking University, 38 Xueyuan Road, Haidian District, Beijing 100191, China

**Keywords:** Long term, Blood pressure variability, Cumulative blood pressure, Intracerebral haemorrhage, Risk prediction, Incremental value

## Abstract

**Aims:**

The relationships between long-term blood pressure (BP) measures and intracerebral haemorrhage (ICH), as well as their predictive ability on ICH, are unclear. In this study, we aim to investigate the independent associations of multiple BP measures with subsequent 5-year ICH risk, as well as the incremental value of these measures over a single-point BP measurement in ICH risk prediction.

**Methods and results:**

We included 12 398 participants from the China Kadoorie Biobank (CKB) who completed three surveys every 4–5 years. The following long-term BP measures were calculated: mean, minimum, maximum, standard deviation, coefficient of variation, average real variability, and cumulative BP exposure (cumBP). Cox proportional hazard models were used to examine the associations between these measures and ICH. The potential incremental value of these measures in ICH risk prediction was assessed using Harrell’s C statistics, continuous net reclassification improvement (cNRI), and relative integrated discrimination improvement (rIDI). The hazard ratios (95% confidence intervals) of incident ICH associated with per standard deviation increase in cumulative systolic BP and cumulative diastolic BP were 1.62 (1.25–2.10) and 1.59 (1.23–2.07), respectively. When cumBP was added to the conventional 5-year ICH risk prediction model, the C-statistic change was 0.009 (−0.001, 0.019), the cNRI was 0.267 (0.070–0.464), and the rIDI was 18.2% (5.8–30.7%). Further subgroup analyses revealed a consistent increase in cNRI and rIDI in men, rural residents, and participants without diabetes. Other long-term BP measures showed no statistically significant associations with incident ICH and generally did not improve model performance.

**Conclusion:**

The nearly 10-year cumBP was positively associated with an increased 5-year risk of ICH and could significantly improve risk reclassification for the ICH risk prediction model that included single-point BP measurement.

**See the editorial comment for this article ‘High blood pressure and intracerebral haemorrhage: a recognized risk factor with new knowledge’, by S. Cinza-Sanjurjo and J.R. González-Juanatey, https://doi.org/10.1093/eurjpc/zwae187.**

## Introduction

Intracerebral haemorrhage (ICH) is one of the most common haemorrhagic stroke subtypes.^1^ In 2019, ICH accounted for 29.5% of all incident strokes in low- and middle-income countries, approximately twice as many as in high-income countries.^2^ Despite accounting for less than one-third of all strokes, the prognosis of ICH is extremely poor, with the case fatality rate as high as 50% at 30 days. Globally, the proportion of stroke deaths attributable to ICH will rise from 44.3% in 2020 to 52.4% in 2050.^3^ Because no effective treatment has been demonstrated to significantly reduce mortality or improve neurological deficits following ICH, current guidelines for ICH management focus primarily on managing risk factors to prevent ICH.^4^

Blood pressure (BP) is one of the most important modifiable risk factors for ICH, and it is more strongly associated with ICH than with subarachnoid haemorrhage (SAH), ischaemic stroke, ischaemic heart disease (IHD), and other vascular diseases.^5–7^ A growing number of studies have found that, in addition to the absolute degree of BP elevation, greater BP variability (BPV) measured over years to decades is associated with a higher risk of cardiovascular disease (CVD).^8–10^ Long-term BPV measures, such as standard deviation (SD), coefficient of variation (CV), average real variability (ARV), and cumulative BP exposure (cumBP), have been linked to all-cause mortality,^9–12^ myocardial infarction,^9,11^ coronary heart disease,^12^ and stroke,^9,11,12^ independent of single-point BP measurement. Existing research, however, has not consistently demonstrated the incremental value of these long-term BPV measures beyond the single-point measurement in predicting the risk of cardiovascular outcomes.^10,13–15^ Furthermore, no study has compared the predictive ability of various BPV measures on specific outcomes using the same population data and a unified statistical strategy.

Previous studies have primarily examined the relationships between single-point BP measurement and ICH, with little use of long-term BPV measures.^7,16,17^ Only two studies conducted in China and The Netherlands found that visit-to-visit BPV was associated with an increased risk of haemorrhagic stroke.^18,19^ These two studies did not distinguish between ICH and SAH. Neither study evaluated the additional value of their respective BPV measures over single-point BP measurement in haemorrhagic stroke risk prediction. More potentially valuable BPV measures, such as cumBP, are warranted to be investigated in relation to ICH, as well as a comparison of the incremental values of various BPV measures in ICH risk prediction.

The current study analysed data from 12 398 Chinese adults who participated in the China Kadoorie Biobank (CKB) and received three surveys every 4–5 years. We aimed to examine the independent associations between multiple long-term BPV measures and the incident risk of ICH. We further assessed the incremental predictive value of these BPV measures over a single-point BP measurement for a 5-year ICH risk prediction model.

## Methods

### Study design and population

During 2004–08, the CKB baseline survey enrolled more than 0.5 million participants aged 30–79 from five urban (Qingdao, Harbin, Liuzhou, Suzhou, and Haikou; represented by the city name) and five rural (Sichuan, Zhejiang, Hunan, Gansu, and Henan; represented by the provincial name) areas across China. About 5% of surviving participants were cluster-sampled at the 2008 and 2013–14 resurveys. The baseline survey and resurvey design were previously described in detail.^20,21^ Shortly after the baseline survey was completed, all participants were followed up for mortality, morbidity, and hospitalization events through linkages to local disease and mortality registries and the national health insurance database, supplemented with annual active confirmation. All events were coded using the International Classification of Diseases, Tenth Revision (ICD-10), by trained staff who were unaware of the baseline information. All participants provided written, informed consent. The study protocol was approved by the Ethics Review Committee of the Chinese Center for Disease Control and Prevention (Beijing, China) and the Oxford Tropical Research Ethics Committee, University of Oxford (UK).

**Figure 1:**
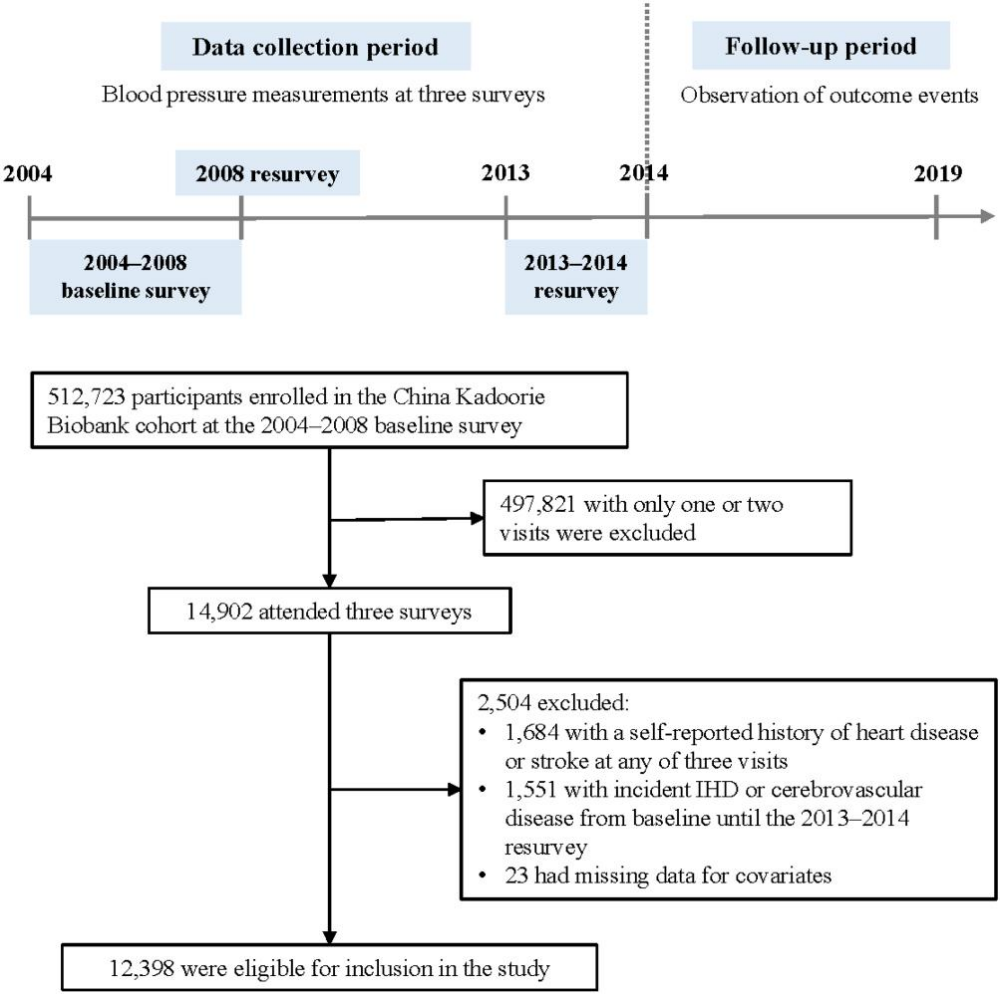
Study design and flow chart of the study participants.

A total of 14 902 participants attended three surveys, namely the 2004–08 baseline survey, the 2008 resurvey, and the 2013–14 resurvey. We excluded participants with a self-reported history of heart disease or stroke at any of three visits (*n* = 1684) or those who were documented with the incidence of IHD (ICD-10: I20–I25) or cerebrovascular disease (I60–I69) from baseline until the 2013–14 resurvey date (*n* = 1551). Participants with missing data for body mass index (BMI; *n* = 1), waist circumference (*n* = 2), and self-reported diseases (*n* = 20) were also excluded, leaving the present study with 12 398 participants (*Figure 1*). The study outcome of interest was the first incident ICH (I61) from the 2013–14 resurvey until 31 December 2018. The adjudication for ICH showed that the positive predictive value was 90.4% for reporting accuracy and 98.2% for diagnostic accuracy.^22^

### Blood pressure measurement and correction

After at least 5 min of resting, all participants had their BP measured twice from the right upper arm by trained staff using a UA-779 digital sphygmomanometer for the 2004–08 baseline survey and 2008 resurvey and Omron HEM-7430 sphygmomanometer for the 2013–14 resurvey. If the difference between two systolic BP (SBP) readings was >10 mmHg, a third measurement was taken after a 1 min rest. The mean values of the last two readings were used for analyses.^6^ Participants were considered to have hypertension if they had self-reported doctor-diagnosed hypertension, were taking anti-hypertensive medication, or had a mean SBP of ≥140 mmHg or a mean diastolic BP (DBP) of ≥90 mmHg.

In a previous study of the CKB population, outdoor temperature was linked to significant seasonal variations in BP.^23^ We, therefore, corrected the measured BP values for seasonal fluctuations in ambient temperature by standardizing them to mid-season (i.e. April) values in each area with reference to a previous study.^24^ Daily ambient temperatures were obtained from the local Meteorological Services during the CKB baseline survey and 2008 resurvey. Real-time temperature was recorded using an on-site thermometer during the 2013–14 resurvey. For consistency, we replaced the daily ambient temperatures at the baseline survey and 2008 resurvey with real-time temperature values (precise to the hour) using meteorological data based on historical reanalysis data sets from the National Aeronautics and Space Administration (NASA), provided by https://xihe-energy.com.^25^ All participants were assigned a temperature value based on the start time of the survey as recorded by the laptop for the survey. Based on the known average treatment efficacy of anti-hypertensive drugs, BP values in treated participants were further corrected by adding 15 mmHg to SBP and 10 mmHg to DBP.^26^

### Other covariate assessments

A laptop-based questionnaire administered by an interviewer was used to collect information on socio-demographic characteristics (e.g. age, sex, level of education, occupation, marital status, and annual household income), lifestyle and dietary factors (e.g. tobacco smoking, alcohol consumption, physical activity, and intake of fresh fruits, vegetables, and red meat), and personal and family medical histories (e.g. heart attack, stroke, and diabetes). The assessment and definition of lifestyle and dietary factors have been described elsewhere.^27–31^ Anthropometric measurements were taken using standard procedures and instruments, including height, weight, and waist circumference. Body mass index was calculated as weight in kilograms divided by height in metres squared. A blood sample of 10 mL was collected for storage and on-site glucose testing. Participants with self-reported diabetes or screen-detected diabetes (a fasting time ≥8 h and a plasma glucose concentration of ≥7.0 mmol/L, or a fasting time <8 h and plasma glucose concentration of ≥11.1 mmol/L) were considered to have diabetes.^32^

### Statistical analysis

First, we calculated the mean, minimum (MIN), maximum (MAX), and SD of three longitudinal SBP and DBP measurements for each participant. The CV was calculated as SD divided by mean SBP or mean DBP. The ARV was the average of the absolute difference between consecutive BP measurements. The cumBP was the area under the curve for three BP measurements and was calculated as:


$$\mathrm{cumBP}=\left(\frac{BP_{1}+BP_{2}}{2}\times T_{12}\right)+\left(\frac{BP_{2}+BP_{3}}{2}\times T_{23}\right)$$

where $BP_{n}$ is the BP value at visit *n* and $T_{ab}$ is the number of years between visits *a* and *b*.

The basic characteristics of the study participants of the 2013–14 resurvey were presented by tertiles of cumSBP and cumDBP. The groups were compared using linear regression for continuous variables and logistic regression for dichotomous variables, with adjustments for age, sex, and 10 study areas as appropriate. The linear trend was tested by treating cumSBP and cumDBP as continuous variables in the model.

Person-years were calculated from the completion of the 2013–14 resurvey to the first diagnosis of ICH, death, loss to follow-up, or 31 December 2018, whichever came first. The Cox proportional hazard model was used to calculate hazard ratios (HRs) and 95% confidence intervals (CIs) for associations of ICH risk with per SD increase in SBP and DBP measures (mean, MIN, MAX, SD, CV, ARV, and cumBP), separately. The models used age as the time scale and were stratified by age (5-year intervals), sex, and study areas (10 groups) as appropriate. Model 1 was adjusted for age (years), education (no formal school, primary school, middle school, high school, technical school or college, and university), occupation (agriculture and related workers, factory worker, administrator/manager, professional/technical, sales and service workers, retired, house wife/husband, self-employed, unemployed, and other/not stated), marital status (married, widowed, separated/divorced, and never married), household income (<¥2500, ¥2500–4999, ¥5000–9999, ¥10 000–19 999, ¥20 000–34 999, ¥35 000–49 999, ¥50 000–74 999, ¥75 000–99 999, and ≥¥100 000), smoking (never, former, and current daily <15, 15–24, or ≥25 cigarettes or equivalent), alcohol drinking (less than weekly, former, weekly, and daily <30, 30–59, or ≥60 g of pure alcohol), intake frequency of fresh fruits, vegetables, and red meat (days/week), total physical activity level (metabolic equivalent task-hour/day), BMI (kg/m^2^), waist circumference (cm), family history of CVD (yes/no), and prevalence of diabetes (yes/no). Model 2 was further adjusted for single-point SBP and DBP. All covariate information was collected from the 2013–14 resurvey. To control the probability of reporting false-positive results for multiple hypothesis tests (*n* = 14), we additionally set the cut-off *α*-value for Bonferroni correction as 0.05/14 = 0.004.^33^

We further evaluated the potential incremental predictive value of long-term BPV measures in the 5-year ICH risk prediction. We first developed a basic risk prediction model for ICH based on our previous CVD risk prediction model.^34^ The Cox model was used for model development and stratified by sex and 10 study areas, with time since 2013–14 resurvey as the time scale. Predictors included age (years), SBP (mmHg), DBP (mmHg), anti-hypertensive treatment (yes/no), current daily smoking (yes/no), history of diabetes (yes/no), and waist circumference (cm), all based on information from the 2013–14 resurvey. The interactions between age and the other six variables were also included. Since the anti-hypertensive treatment was one of the predictors, no additional 15/10 mmHg was added to SBP and DBP. We additionally added the CV, ARV, mean + SD + MIN + MAX, and cumBP to the basic model separately. The model’s discrimination ability was assessed by Harrell’s C statistics.^35^ We calculated the 95% CIs for C statistics and the difference in C-statistic values between the above four models and the basic model by bootstrapping. A prior power analysis on all participants indicated that a power of 80% could be achieved with a C-statistic change of at least 0.003. The continuous net reclassification improvement (cNRI) and the relative integrated discrimination improvement (rIDI) were also used to assess the reclassification performance of the models.^36,37^ Analyses were performed on all participants and also stratified by age (≥65 and <65 years), sex (men and women), area of residence (rural and urban), and prevalence of diabetes (yes/no). We only presented results from participants without diabetes at the 2013–14 resurvey because the number of participants with diabetes was small.

We conducted several sensitivity analyses: (i) adjusted for self-reported anti-hypertensive treatment at each visit as covariates instead of adding 15/10 mmHg to SBP/DBP; (ii) additionally adjusted for heart rate variability (SD) and incorporated it into the basic prediction model; and (iii) additionally adjusted for menopausal status at 2013–14 resurvey and incorporated it into the basic prediction model for women.

The power analysis was performed using PASS (15.0.5). All other analyses were performed using Stata (version 15.0). Two-sided *P*-values of <0.05 were considered statistically significant.

## Results

### Background characteristics of study participants

The mean (SD) age of the 12 398 participants was 50.3 (9.8) in the 2004–08 baseline survey and 58.4 (9.9) in the 2013–14 resurvey. Women made up 62.1% of the participants, and 64.9% lived in rural areas. Between the 2004–08 baseline survey and the 2013–14 resurvey, the average SBP (SD) increased from 131.3 (19.8) to 136.6 (20.2) mmHg, the average DBP (SD) increased from 77.6 (10.8) to 78.6 (11.0) mmHg, and the prevalence of hypertension increased from 31.4 to 52.2%. *Table 1* and Supplementary material online, *Table S1* display the basic characteristics of participants based on cumSBP and cumDBP tertiles. Participants with higher cumSBP and cumDBP were more likely to be older, live in rural areas, have a higher BMI and waist circumference, and have a higher prevalence of daily alcohol drinking and diabetes.

**Table 1:** Basic characteristics of participants by tertiles of cumulative blood pressure

	Tertile of cumSBP				Tertile of cumDBP			
	Low	Medium	High	*P*-value for trend	Low	Medium	High	*P*-value for trend
Age at 2004–08 baseline, years	47.1 (9.0)	49.7 (9.5)	54.0 (9.7)	<0.001	49.5 (10.3)	50.2 (9.8)	51.1 (9.4)	<0.001
Age at 2013–14 resurvey, years	54.7 (9.0)	57.8 (9.4)	62.6 (9.6)	<0.001	57.1 (10.3)	58.3 (9.8)	59.6 (9.4)	<0.001
Women, %	65.4	59.0	62.0	0.826	65.2	61.2	59.9	<0.001
Menopausal women, %	42.0	45.2	55.7	<0.001	45.7	46.9	50.3	<0.001
Rural, %	56.4	66.3	72.1	<0.001	56.2	65.6	72.8	<0.001
Middle school or above, %	45.3	44.9	44.7	0.528	45.0	44.6	45.3	0.980
Agriculture and related workers, %	34.9	31.9	30.2	<0.001	34.8	31.6	30.6	<0.001
Married, %	88.9	88.5	88.4	0.248	88.8	88.0	88.9	0.953
Household income ≥¥50 000, %	44.1	44.2	43.0	0.316	43.8	43.2	44.5	0.918
Current daily smoking, %	22.6	20.7	19.7	<0.001	22.6	20.4	20.1	<0.001
Current daily alcohol drinking, %	8.3	9.1	9.9	0.014	8.2	9.4	9.8	0.002
*Daily food consumption, %*								
Fresh fruits	31.3	31.9	30.8	0.595	31.3	31.3	31.5	0.735
Fresh vegetables	95.8	96.4	96.8	0.006	95.8	96.4	96.9	0.001
Red meat	42.6	42.7	41.0	0.043	42.5	42.3	41.5	0.304
Total physical activity, MET-h/day	19.1 (13.9)	19.2 (14.1)	18.6 (13.6)	0.003	19.5 (13.7)	18.9 (14.0)	18.5 (14.2)	<0.001
Body mass index, kg/m²	23.0 (3.2)	24.1 (3.4)	24.9 (3.6)	<0.001	23.1 (3.3)	24.0 (3.3)	24.9 (3.6)	<0.001
Waist circumference, cm	81.2 (9.2)	84.2 (9.5)	86.3 (10.2)	<0.001	81.3 (9.4)	84.1 (9.3)	86.4 (10.1)	<0.001
Diabetes, %	5.6	9.2	14.2	<0.001	7.1	9.6	12.6	<0.001
Hypertension, %	16.8	51.8	89.4	<0.001	24.3	50.1	82.4	<0.001
Anti-hypertensive treatment, %	2.5	12.8	44.8	<0.001	4.3	14.5	42.6	<0.001
Family history of cardiovascular disease, %	20.3	23.3	24.9	<0.001	20.3	22.5	25.7	<0.001
SBP at 2004–08 baseline, mmHg	119.2 (12.4)	128.7 (13.8)	145.9 (19.9)	<0.001	121.6 (14.6)	129.6 (16.4)	142.6 (20.8)	<0.001
SBP at 2013–14 resurvey, mmHg	122.7 (12.7)	135.4 (14.9)	151.7 (19.2)	<0.001	125.8 (16.3)	136.3 (17.5)	147.7 (19.7)	<0.001
DBP at 2004–08 baseline, mmHg	71.4 (8.4)	76.8 (9.0)	84.6 (11.0)	<0.001	70.1 (7.9)	77.1 (8.2)	85.6 (10.2)	<0.001
DBP at 2013–14 resurvey, mmHg	72.3 (8.8)	78.5 (9.9)	84.9 (11.4)	<0.001	70.7 (8.2)	78.6 (8.6)	86.5 (10.3)	<0.001
cumSBP, mmHg × year	905.7 (66.3)	1065.7 (45.4)	1298.1 (135.2)	—	933.6 (104.2)	1071.1 (106.6)	1264.9 (158.9)	<0.001
cumDBP, mmHg × year	540.8 (52.7)	630.2 (55.9)	743.5 (86.8)	<0.001	532.5 (41.3)	628.8 (26.1)	753.1 (68.9)	—

The basic characteristics at the 2013–14 resurvey are presented in the table unless otherwise stated. Except for age, women, menopausal women, and urban variables, data were presented as mean (SD) or percentage, with adjustments for age, sex, and study areas.

cumDBP, cumulative diastolic blood pressure; cumSBP, cumulative systolic blood pressure; MET-h/d, metabolic equivalent task-hour/day.

### Associations between long-term blood pressure measures and intracerebral haemorrhage

Since the 2013–14 resurvey, incident ICH has occurred in 121 participants during a median follow-up of 5 years (59 653 person-years), with an incidence rate of 2.03 per 1000 person-years. After controlling for potential confounders, all long-term SBP measures from the 2004–08 baseline survey to the 2013–14 resurvey were associated with an increased risk of ICH (Model 1 in *Table 2*). When further adjustments for single-point SBP and DBP from the 2013–14 resurvey were made, all effect sizes were attenuated (Model 2). Nevertheless, associations between 5 long-term SBP measures and ICH persisted except for the Mean and MIN. The HRs (95% CIs) of incident ICH associated with per SD increment in SBP measures were 1.65 (1.15–2.38) for MAX, 1.23 (1.03–1.48) for SD, 1.20 (1.01–1.43) for CV, 1.20 (1.01–1.42) for ARV, and 1.62 (1.25–2.10) for cumSBP. The MAX and cumDBP were the only DBP measures associated with an increased risk of ICH, with HRs of 1.54 (1.08–2.19) and 1.59 (1.23–2.07) for each SD increase, respectively. The cumSBP and cumDBP remained statistically significant after Bonferroni correction at a *P*-value of <0.004.

**Table 2:** Associations between per 1 standard deviation increment in blood pressure measures and incident intracerebral haemorrhage

	Model 1	Model 2	*P*-value^a^
*SBP*			
Mean, per 19.5 mmHg	1.81 (1.52–2.16)	1.25 (0.87–1.79)	0.226
MIN, per 17.8 mmHg	1.58 (1.33–1.88)	0.96 (0.74–1.25)	0.774
MAX, per 23.3 mmHg	1.92 (1.61–2.28)	1.65 (1.15–2.38)	0.007
SD, per 6.8 mmHg	1.52 (1.30–1.77)	1.23 (1.03–1.48)	0.026
CV, per 4.5%	1.40 (1.19–1.64)	1.20 (1.01–1.43)	0.039
ARV, per 8.4 mmHg	1.45 (1.26–1.68)	1.20 (1.01–1.42)	0.040
cumSBP, per 191.9 mmHg × year	2.01 (1.68–2.41)	1.62 (1.25–2.10)	<0.001
*DBP*			
Mean, per 10.4 mmHg	1.81 (1.52–2.15)	1.26 (0.89–1.79)	0.188
MIN, per 10.2 mmHg	1.65 (1.38–1.97)	1.04 (0.79–1.35)	0.791
MAX, per 11.8 mmHg	1.85 (1.56–2.19)	1.54 (1.08–2.19)	0.018
SD, per 3.6 mmHg	1.36 (1.16–1.58)	1.14 (0.96–1.36)	0.133
CV, per 4.4%	1.21 (1.04–1.42)	1.11 (0.94–1.32)	0.213
ARV, per 4.6 mmHg	1.31 (1.12–1.54)	1.11 (0.93–1.33)	0.240
cumDBP, per 104.4 mmHg × year	1.97 (1.65–2.36)	1.59 (1.23–2.07)	<0.001

The HRs and 95% CIs are presented in the table, with the Cox model stratified by age (5-year intervals), sex, and study areas (10 groups). Model 1 was adjusted for age, education, occupation, marital status, household income, smoking, alcohol drinking, intake frequency of fresh fruits, vegetables, and red meat, total physical activity level, BMI, waist circumference, family history of CVD, and prevalence of diabetes, all from the 2013–14 resurvey. Model 2 was further adjusted for single-point SBP and DBP at 2013–14 resurvey.

ARV, average real variability; cumDBP, cumulative diastolic blood pressure; cumSBP, cumulative systolic blood pressure; CV, coefficient of variation; MAX, maximum; MIN, minimum; SD, standard deviation.

^a^This column displays the *P*-values of Model 2. The cut-off *α*-value for Bonferroni correction = 0.05/14 = 0.004.

Stratification analyses revealed that cumSBP and cumDBP were associated with an increased risk of ICH in all age groups, men, rural residents, and participants without diabetes (Model 2 in Supplementary material online, *Tables S2*–*S5*). Only in individual subgroups did other SBP and DBP measures show statistically significant associations with ICH. Positive associations between cumBP and ICH survived Bonferroni correction in men, rural residents, and participants without diabetes. Using self-reported anti-hypertensive treatment as a covariate rather than adding 15/10 mmHg to SBP/DBP yielded generally consistent results (data not provided). Sensitivity analyses that additionally adjusted for heart rate variability or female menopausal status did not alter the results substantially (see Supplementary material online, *Tables S6* and *S7*).

### Discrimination performance of prediction models

**Figure 2:**
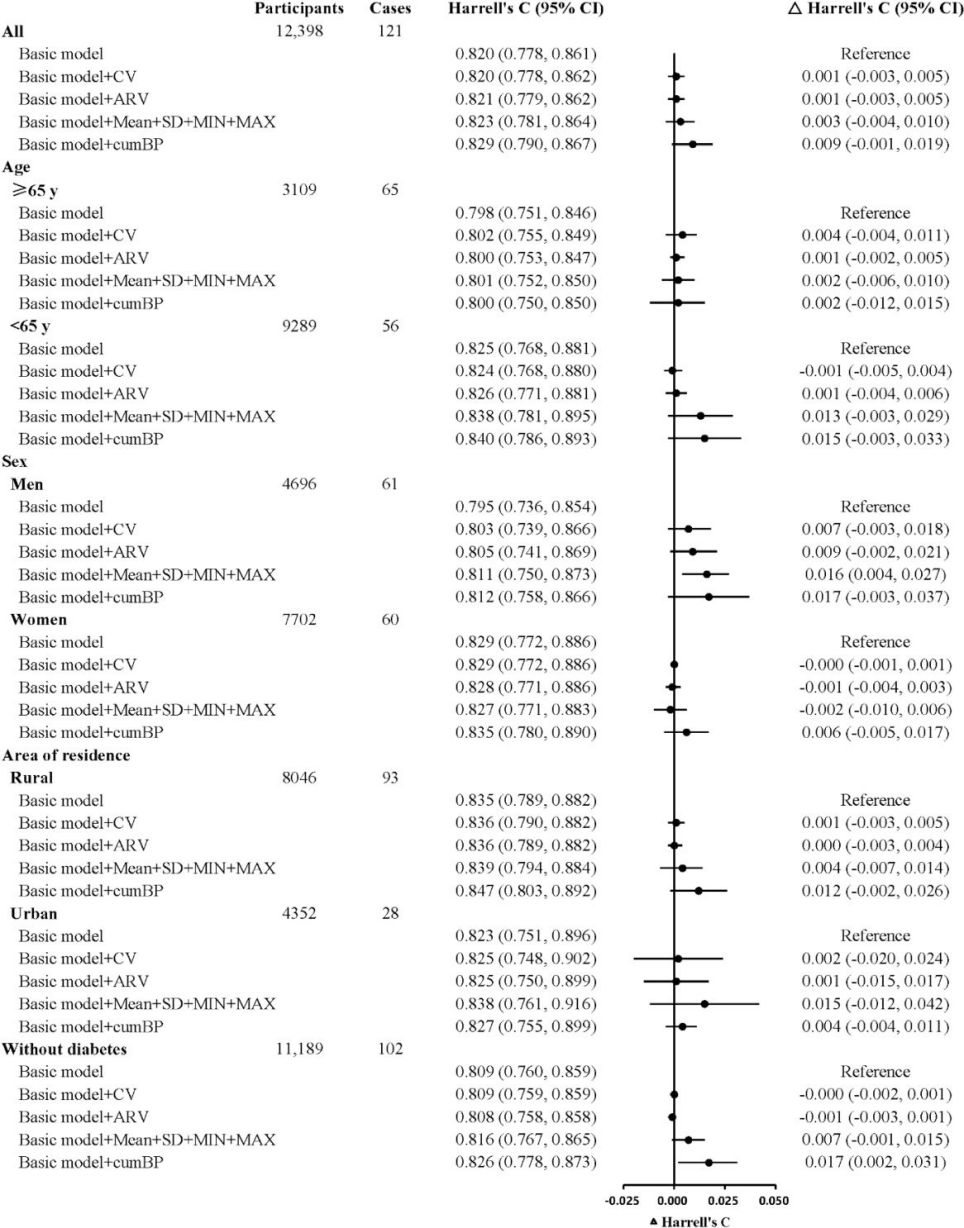
Harrell’s C change for 5-year intracerebral haemorrhage risk prediction models by including both systolic blood pressure and diastolic blood pressure measures in all participants and by age, sex, area of residence, or prevalence of diabetes. Basic Cox models included age, systolic blood pressure, diastolic blood pressure, anti-hypertensive treatment, current daily smoking, prevalence of diabetes, and waist circumference at 2013–14 resurvey, and interactions between age and the other six predictors. For the analysis in participants without diabetes, the prevalence of diabetes was removed from the basic model. All models were stratified by sex and 10 study areas, except for the analysis by sex, in which the model was stratified only by 10 study areas. ARV, average real variability; CI, confidence interval; cumBP, cumulative blood pressure; CV, coefficient of variation; MAX, maximum; MIN, minimum; SD, standard deviation.

When the CV or ARV of both SBP and DBP were included in the conventional 5-year ICH risk prediction models, no statistically significant improvement in C-statistic was observed in either all participants or subgroups (*Figure 2*). Adding Mean + SD + MIN + MAX of both SBP and DBP improved C-statistic only in men, with an increase (95% CI) of 0.016 (0.004–0.027).

The addition of both cumSBP and cumDBP resulted in a marginal improvement in model performance for all participants, with a C-statistic change of 0.009 (−0.001, 0.019). When restricted to participants without diabetes, adding cumBP to the model resulted in a moderate discriminative improvement, with a C-statistic increase of 0.017 (0.002–0.031). There were no statistically significant increases in the C-statistic in the other subgroups. Sensitivity analyses showed consistent results (see Supplementary material online, *Tables S8* and *S9*).

### Reclassification performance of prediction models

In the analyses of all participants and subgroups, most of the additions of CV or ARV to conventional 5-year ICH risk prediction models did not improve the model reclassification performance. Only adding ARV resulted in a slight improvement in cNRI in men (*Table 3*). There was no improvement in cNRI or rIDI with the Mean + SD + MIN + MAX of both SBP and DBP for all participants. However, cNRI and rIDI increased in certain sex or area of residence subgroups, with cNRIs (95% CI) of 0.294 (0.038–0.551) and 0.621 (0.241–1.001) for men and urban residents, and rIDIs (95% CI) of 20.0% (4.0–36.1%) and 20.1% (4.5–35.7%) for women and rural residents, respectively.

**Table 3:** Added predictive ability of both systolic and diastolic blood pressure measures for 5-year intracerebral haemorrhage risk in all participants and by age, sex, area of residence, or prevalence of diabetes

	cNRI (95% CI)	rIDI (%) (95% CI)
*All*		
Basic model + CV	−0.016 (−0.216, 0.185)	5.2 (−1.1, 11.5)
Basic model + ARV	0.026 (−0.164, 0.216)	2.0 (−3.0, 6.9)
Basic model + Mean + SD + MIN + MAX	0.140 (−0.066, 0.347)	11.8 (−2.7, 26.3)
Basic model + cumBP	0.267 (0.070–0.464)	18.2 (5.8–30.7)
Age		
*≥65 years*		
Basic model + CV	0.103 (−0.141, 0.347)	6.6 (−2.3, 15.5)
Basic model + ARV	0.018 (−0.225, 0.261)	1.4 (−3.2, 5.9)
Basic model + Mean + SD + MIN + MAX	0.160 (−0.083, 0.403)	9.0 (−1.3, 19.3)
Basic model + cumBP	0.171 (−0.109, 0.450)	15.6 (4.8–26.5)
*<65 years*		
Basic model + CV	0.045 (−0.250, 0.339)	3.4 (−3.8, 10.5)
Basic model + ARV	0.088 (−0.187, 0.362)	4.7 (−4.2, 13.6)
Basic model + Mean + SD + MIN + MAX	0.202 (−0.074, 0.478)	15.6 (−3.3, 34.6)
Basic model + cumBP	0.225 (−0.048, 0.498)	15.7 (−7.0, 38.4)
Sex		
*Men*		
Basic model + CV	0.126 (−0.181, 0.432)	2.2 (−17.8, 22.2)
Basic model + ARV	0.281 (0.001–0.562)	−0.0 (−19.0, 18.9)
Basic model + Mean + SD + MIN + MAX	0.294 (0.038–0.551)	1.3 (−20.3, 23.0)
Basic model + cumBP	0.325 (0.067–0.583)	19.2 (0.4–38.0)
*Women*		
Basic model + CV	−0.064 (−0.337, 0.208)	0.6 (−0.3, 1.5)
Basic model + ARV	0.151 (−0.139, 0.441)	−0.1 (−2.6, 2.4)
Basic model + Mean + SD + MIN + MAX	−0.008 (−0.240, 0.225)	20.0 (4.0–36.1)
Basic model + cumBP	0.147 (−0.098, 0.392)	18.8 (2.7–34.8)
Area of residence		
*Rural*		
Basic model + CV	−0.005 (−0.225, 0.215)	4.2 (−2.1, 10.5)
Basic model + ARV	0.076 (−0.118, 0.271)	3.9 (−2.1, 9.8)
Basic model + Mean + SD + MIN + MAX	0.087 (−0.123, 0.297)	20.1 (4.5–35.7)
Basic model + cumBP	0.271 (0.037–0.504)	20.2 (2.9–37.4)
*Urban*		
Basic model + CV	0.372 (−0.037, 0.781)	20.9 (−16.9, 58.8)
Basic model + ARV	0.272 (−0.131, 0.675)	11.2 (−27.8, 50.1)
Basic model + Mean + SD + MIN + MAX	0.621 (0.241–1.001)	33.2 (−32.0, 98.5)
Basic model + cumBP	0.257 (−0.119, 0.633)	2.8 (−10.7, 16.2)
*Without diabetes*		
Basic model + CV	−0.069 (−0.250, 0.113)	0.5 (−2.3, 3.2)
Basic model + ARV	0.027 (−0.175, 0.230)	0.8 (−1.5, 3.1)
Basic model + Mean + SD + MIN + MAX	0.174 (−0.028, 0.376)	10.7 (−6.2, 27.5)
Basic model + cumBP	0.372 (0.157–0.587)	26.2 (3.5–48.9)

Basic Cox models included age, SBP, DBP, anti-hypertensive treatment, current daily smoking, prevalence of diabetes, and waist circumference at 2013–14 resurvey, and interactions between age and the other six predictors. For the analysis in participants without diabetes, the prevalence of diabetes was removed from the basic model. All models were stratified by sex and 10 study areas, except for the analysis by sex in which the model was only stratified by 10 study areas.

ARV, average real variability; CI, confidence interval; cNRI, continuous net reclassification improvement; cumBP, cumulative blood pressure; CV, coefficient of variation; MAX, maximum; MIN, minimum; rIDI, relative integrated discrimination improvement; SD, standard deviation.

When cumSBP and cumDBP were added to the model, the consistently increased cNRI and rIDI indicated that the model’s reclassification capacity was improved for all participants, as well as for men, rural residents, and participants without diabetes. The largest cNRI and rIDI values were seen for participants without diabetes, with 0.372 (0.157–0.587) and 26.2% (3.5–48.9%), respectively. The increased rIDI alone also suggested improvement in model performance due to the inclusion of cumBP for women and participants aged 65 and over. Sensitivity analyses showed consistent results (see Supplementary material online, *Tables S8* and *S9*).

## Discussion

In the current cohort study of 12 398 Chinese adults, the cumBP, based on three repeatedly measured BPs every 4–5 years, was positively associated with subsequent 5-year risk of ICH, independent of the most recent single-point SBP and DBP. The cumBP could improve the risk reclassification of the conventional 5-year ICH risk prediction model that included single-point BP measurement for all participants, as well as for men, rural residents, and participants without diabetes. Other long-term BPV measures, in comparison, showed no associations with incident ICH and generally failed to provide incremental benefits in predicting ICH risk, despite slight improvements in risk reclassification in individual subgroups.

The cumBP has been identified as a marker that allows for a more comprehensive assessment of the duration and intensity of long-term BP exposure. Evidence from Western populations indicated that the cumBP was associated with incident stroke^12,38^ and predicted CVD risk.^13,38^ The Lifetime Risk Pooling Project of American participants aged 45–60 found that every 130 mmHg × year increase in 10-year cumSBP was associated with a 33% increase in the subsequent 12.9-year risk of stroke after adjusting for the most recent single-point SBP and other covariates.^12^ Our study also found robust and strong relationships between almost 10-year cumBP and subsequent 5-year ICH risk, with each SD increase in cumSBP (192 mmHg × year) and cumDBP (104 mmHg × year) associated with increased ICH risks by 62 and 59%, respectively.

One of the primary goals of this study is to assess the incremental value of cumBP and other long-term BPV measures for the conventional ICH risk prediction model with single-point BP measurement. We did not find a statistically significant improvement in the C-statistic when cumSBP and cumDBP were added to the conventional model. However, the C-statistic is a conservative method for assessing changes in model fit and hardly moves once some good risk factors have been included in the model.^36^ Using the widely used NRI and IDI,^37^ we found that adding cumBP could significantly improve the reclassification ability of the overall participant model. The cumBP, in particular, provided a significant incremental benefit in men, rural residents, and participants without diabetes. The efforts to discover new useful biomarkers that could improve risk prediction are generally aimed at the entire population. Perhaps we can direct our efforts towards specific subpopulations.

Using a unified statistical strategy, all BPV measures, except cumBP, had no statistically significant associations with incident ICH after Bonferroni correction. Furthermore, these measures added little to the predictive performance of the conventional 5-year ICH risk prediction model. Certain measures, such as Mean + SD + MIN + MAX, demonstrated statistically significant increases in cNRI or rIDI in individual subgroups but lacked consistency and robustness overall. A Kailuan study of over 50 000 Chinese adults, aged 53 on average at baseline, examined the associations between visit-to-visit BPV and subsequent 3-year risk of stroke, as well as its subtypes (ischaemic and haemorrhagic stroke). The SD, CV, and ARV were calculated using repeatedly measured BPs over three biennial visits. After controlling for mean BP level and other potential confounders, each SD increase in SBP measures was associated with a 26% increase in the risk of haemorrhagic stroke for SD (6.7 mmHg), a 34% increase for CV (4.7%), and a 20% increase for ARV (9.2 mmHg), respectively. The corresponding increased risk of haemorrhagic stroke associated with DBP measures was 28% for SD (4.1 mmHg) and 30% for CV (4.7%), with no association observed for ARV.^18^ Similar associations of ICH with SD, CV, and ARV of SBP were observed in our study before Bonferroni correction, whereas no statistical associations were found for the corresponding DBP measures. Aside from differences in geographic and socio-demographic characteristics, the baseline prevalence of hypertension in the Kailuan study was 44%, which was significantly higher than the 31% in our population. In hypertensive individuals, a larger long-term variation in DBP may have a greater impact on stroke.

To the best of our knowledge, this is the first prospective study that used the same population data and a unified statistical strategy to compare the independent relationships between multiple long-term BPV measures and incident ICH, as well as to assess the potential utility of these measures in the ICH risk prediction model beyond single-point BP measurement. We used data from three regular surveys, as well as a subsequent 5-year outcome surveillance period, for a total of nearly 15 years of observation. This study included participants from 10 geographically diverse urban and rural areas in China, representing various socio-demographic characteristics. We could distinguish two haemorrhagic stroke subtypes: ICH and SAH. Due to a small number of cases, the analysis of SAH was not included. Furthermore, comprehensive information collection from the questionnaire and anthropometric measurements aided in the control of potential confounders.

Several limitations of our study merit consideration. First, BP fluctuations over a shorter period (weeks, months, 1 or 2 years) were not captured. Furthermore, there were only three time points in this study, preventing us from conducting a more in-depth investigation into how long the BP variation was sufficient to predict the subsequent ICH risk. Second, our study population was restricted to those who completed three surveys and had no history of CVD from baseline to the 2013–14 resurvey, which may limit generalizability to those who developed CVD or died prematurely. Nonetheless, these excluded participants may be at a higher risk of CVD and would most likely be recognized by the conventional model based on single-point BP measurement. We emphasized the additional value of long-term BPV rather than substituting for single-point absolute BP level in the conventional model. Third, there may be residual confounding because we did not collect data on personal habits, such as high-salt diets, staying up late, and overworking. Finally, the BP measurements in this study followed guidelines at the time of the surveys.^39,40^ However, when compared with the updated guideline requirements in recent years, which require taking three measurements with a 1 min interval between each and averaging the last two measurements, our accuracy may be compromised to some extent.

This prospective cohort study of Chinese adults found that the nearly 10-year cumBP was positively associated with an increased subsequent 5-year risk of ICH and could significantly improve risk reclassification for the ICH risk prediction model that included single-point BP measurement. The ICH continues to impose a heavy burden on China. Blood pressure is now a routine measurement for regular health check-ups, establishment and management of resident health records in the Basic Public Health Services project, and hospital visits. The advancement of electronic medical records makes it easier to obtain long-term repeated BP measurement data. To better identify potential high-risk groups for ICH for early intervention, it is necessary to consider BP measured multiple times in the past and use appropriate indicators, such as cumBP, in risk prediction.

## Supplementary Material

zwae147_Supplementary_Data

## Data Availability

The CKB is a global resource for the investigation of lifestyle, environmental, blood biochemical, and genetic factors as determinants of common diseases. The CKB study group is committed to making the cohort data available to the scientific community in China, the UK, and worldwide to advance knowledge about the causes, prevention, and treatment of the disease. For detailed information on what data are currently available to open access users and how to apply for it, visit: https://www.ckbiobank.org/data-access. Researchers who are interested in obtaining the raw data from the CKB study that underlines this paper should contact ckbaccess@ndph.ox.ac.uk. A research proposal will be requested to ensure that any analysis is performed by bona fide researchers and—where data are not currently available to open access researchers—is restricted to the topic covered in this paper.
