# Supplementary material for: Predictive value of 8-year blood pressure measures in intracerebral haemorrhage risk over 5 years
Source: Eur J Prev Cardiol. 2024 Apr 17;31(14):1702–10. doi: 10.1093/eurjpc/zwae147 (PMC7616516; doi:10.1093/eurjpc/zwae147)
Supplement: zwae147_Supplementary_Data [file zwae147_supplementary_data.docx]

**Supplementary materials**

Supplementary materials for the manuscript entitled ***Predictive value of 8-year blood pressure measures in intracerebral hemorrhage risk over 5 years***

**Contents**

[**Members of the China Kadoorie Biobank collaborative group:** 1](#_Toc159878220)

[**Table S1.** Baseline characteristics of participants by tertiles of cumulative blood pressure 3](#_Toc159878221)

[**Table S2.** Associations between per 1-SD increment in blood pressure measures and incident intracerebral haemorrhage by age 4](#_Toc159878222)

[**Table S3.** Associations between per 1-SD increment in blood pressure measures and incident intracerebral haemorrhage by sex 5](#_Toc159878223)

[**Table S4.** Associations between per 1-SD increment in blood pressure measures and incident intracerebral haemorrhage by area of residence 6](#_Toc159878224)

[**Table S5.** Associations between per 1-SD increment in blood pressure measures and incident intracerebral haemorrhage in participants without diabetes 7](#_Toc159878225)

[**Table S6.** Sensitivity analysis of the associations between per 1-SD increment in blood pressure measures and incident intracerebral hemorrhage 8](#_Toc159878226)

[**Table S7.** Sensitivity analysis of associations between per 1-SD increment in blood pressure measures and incident intracerebral haemorrhage for women 9](#_Toc159878227)

[**Table S8.** Sensitivity analysis of the incremental predictive performance of long-term BP measures for 5-year intracerebral hemorrhage risk 10](#_Toc159878228)

[**Table S9.** Sensitivity analysis of the incremental predictive performance of long-term BP measures for 5-year intracerebral hemorrhage risk for women 11](#_Toc159878229)

**Members of the China Kadoorie Biobank collaborative group:**

**International Steering Committee:** Junshi Chen, Zhengming Chen (PI), Robert Clarke, Rory Collins, Liming Li (PI), Jun Lv, Richard Peto, Robin Walters.

**International Co-ordinating Centre, Oxford:** Daniel Avery, Maxim Barnard, Derrick Bennett, Lazaros Belbasis, Ruth Boxall, Ka Hung Chan, Yiping Chen, Zhengming Chen, Charlotte Clarke, Johnathan Clarke; Robert Clarke, Huaidong Du, Ahmed Edris Mohamed, Hannah Fry, Simon Gilbert, Pek Kei Im, Andri Iona, Maria Kakkoura, Christiana Kartsonaki, Hubert Lam, Kuang Lin, James Liu, Mohsen Mazidi, Iona Millwood, Sam Morris, Qunhua Nie, Alfred Pozarickij, Maryanm Rahmati, Paul Ryder, Saredo Said, Dan Schmidt, Becky Stevens, Iain Turnbull, Robin Walters, Baihan Wang, Lin Wang, Neil Wright, Ling Yang, Xiaoming Yang, Pang Yao.

**National Co-ordinating Centre, Beijing:** Xiao Han, Can Hou, Qingmei Xia, Chao Liu, Jun Lv, Pei Pei, Dianjianyi Sun, Canqing Yu, Lang Pan.

**10 Regional Co-ordinating Centres:**

**Qingdao CDC:** Zengchang Pang, Ruqin Gao, Shanpeng Li, Haiping Duan, Shaojie Wang, Yongmei Liu, Ranran Du, Yajing Zang, Liang Cheng, Xiaocao Tian, Hua Zhang, Yaoming Zhai, Feng Ning, Xiaohui Sun, Feifei Li. **Licang CDC:** Silu Lv, Junzheng Wang, Wei Hou. **Heilongjiang Provincial CDC:** Wei Sun, Shichun Yan, Xiaoming Cui. **Nangang CDC:** Chi Wang, Zhenyuan Wu,Yanjie Li, Quan Kang. **Hainan Provincial CDC:** Huiming Luo, Tingting Ou. **Meilan CDC:** Xiangyang Zheng, Zhendong Guo, Shukuan Wu, Yilei Li, Huimei Li. **Jiangsu Provincial CDC:** Ming Wu, Yonglin Zhou, Jinyi Zhou, Ran Tao, Jie Yang, Jian Su. **Suzhou CDC:** Fang Liu, Jun Zhang, Yihe Hu, Yan Lu, Liangcai Ma, Aiyu Tang, Shuo Zhang, Jianrong Jin, Jingchao Liu. **Guangxi Provincial CDC:** Mei Lin, Zhenzhen Lu. **Liuzhou CDC:** Lifang Zhou, Changping Xie, Jian Lan,Tingping Zhu,Yun Liu, Liuping Wei, Liyuan Zhou, Ningyu Chen, Yulu Qin, Sisi Wang. **Sichuan Provincial CDC:** Xianping Wu, Ningmei Zhang, Xiaofang Chen, Xiaoyu Chang. **Pengzhou CDC:** Mingqiang Yuan, Xia Wu, Xiaofang Chen, Wei Jiang, Jiaqiu Liu, Qiang Sun. **Gansu Provincial CDC:** Faqing Chen, Xiaolan Ren, Caixia Dong. **Maiji CDC:** Hui Zhang, Enke Mao, Xiaoping Wang, Tao Wang, Xi zhang. **Henan Provincial CDC:** Kai Kang, Shixian Feng, Huizi Tian, Lei Fan. **Huixian CDC:** XiaoLin Li, Huarong Sun, Pan He, Xukui Zhang. **Zhejiang Provincial CDC:** Min Yu, Ruying Hu, Hao Wang. **Tongxiang CDC:** Xiaoyi Zhang, Yuan Cao, Kaixu Xie, Lingli Chen, Dun Shen. **Hunan Provincial CDC:** Xiaojun Li, Donghui Jin, Li Yin, Huilin Liu, Zhongxi Fu. **Liuyang CDC:** Xin Xu, Hao Zhang, Jianwei Chen,Yuan Peng, Libo Zhang, Chan Qu.

**Event Adjudication Clinicians:**

**Beijing Tiantan Hospital,Capital Medical University** Shuya Li, Haiqiang Qin, Yongjun Wang, **Peking University People's Hospital** Qiling Chen, Jihua Wang, **The 1st Affiliated Hospital of Harbin Medical University** Xiaojia Sun, Lei Wang, Xun Wang, Liming Zhang, Shanshan Zhou, **The 2nd Affiliated Hospital of Harbin Medical University** Hongyuan Chen, Li Chen, Haiyan Gou, Weizhi Wang, Yanmei Zhu, Yulan Zhu, **The 2nd Hospital of Hebei Medical University** Ning Zhang, **Huashan Hospital** Xin Cheng, Qiang Dong, Yi Dong, Kun Fang, Yiting Mao, **Jinling Hospital** Yu An, Peiling Chen, Yinghua Chen, Zhihong Liu, Lihua Zhang **The People's Hospital of Liaoning Province** Xiaohong Chen, Naixin Jv, Xiaojiu Li, Liyang Liu, Yun Lu, Xiaona Xing, **Qingdao Fuwai Cardiovascular Hospital** Shihao You, **Shengjing Hospital of China Medical University** Xiaoli Cheng, Chaojun Gua, Jinping Jiang, Jingyi Liu, Shumei Ma, **Shenyang Military General Hospital** Xuefeng Yang, **The First People's Hospital of Shenyang** Xiaomo Du, Jian Xu, Xuecheng Yang, Xiaodi Zhao, **West China Hospital, Sichuan University** Zilong Hao, Ming Liu, Deren Wang, **The Second Affiliated Hospital of Suzhou University** Xiaoting Li, **Suzhou Kowloon Hospital Shanghai Jiao Tong University School of Medicine** Lili Hui, Zhanling Liao, Feng Liu, **Qingdao Fuwai Cardiovascular Hospital** Chunning Feng, Dejiang Ji, Fengxia Qu, Wenwen Yuan, **The First Affiliated Hospital of Zhengzhou University** Xin Fu, **Zhongshan Hospital** Jing Ding, Peng Du, Lirong Jin, Yueshi Mao, Xin Wang.

**Table S1.** Baseline characteristics of participants by tertiles of cumulative blood pressure

|  | Tertile of cumSBP | | | | Tertile of cumDBP | | | |
| --- | --- | --- | --- | --- | --- | --- | --- | --- |
|  | Low | Medium | High | P value for trend | Low | Medium | High | P value for trend |
| Middle school or above, % | 45.9 | 45.1 | 43.9 | 0.001 | 46.0 | 45.3 | 43.8 | 0.010 |
| Agriculture and related workers, % | 57.2 | 56.1 | 58.9 | 0.241 | 57.5 | 56.9 | 58.1 | 0.131 |
| Married, % | 93.3 | 93.3 | 92.9 | 0.462 | 93.1 | 93.3 | 93.0 | 0.945 |
| Household income≥¥ 20,000, % | 41.6 | 38.0 | 31.6 | <0.001 | 41.3 | 36.7 | 33.0 | <0.001 |
| Current daily smoking, % | 26.1 | 24.8 | 23.9 | <0.001 | 26.5 | 24.1 | 24.3 | <0.001 |
| Current daily alcohol drinking, % | 7.1 | 8.6 | 9.8 | <0.001 | 7.1 | 8.1 | 10.3 | <0.001 |
| Daily food consumption, % |  |  |  |  |  |  |  |  |
| Fresh fruits | 15.4 | 15.0 | 14.6 | 0.515 | 14.6 | 15.5 | 15.1 | 0.772 |
| Fresh vegetables | 95.4 | 96.3 | 95.7 | 0.333 | 95.6 | 96.2 | 95.6 | 0.455 |
| Red meat | 28.8 | 26.7 | 26.4 | 0.009 | 28.1 | 27.1 | 26.7 | 0.066 |
| Total physical activity, MET-h/day | 22.7 (13.7) | 22.7 (14.7) | 22.5 (15.0) | 0.038 | 22.9 (13.6) | 22.5 (14.5) | 22.5 (15.2) | 0.056 |
| Body mass index, kg/m² | 22.6 (3.0) | 23.6 (3.2) | 24.4 (3.4) | <0.001 | 22.6 (3.1) | 23.6 (3.2) | 24.4 (3.3) | <0.001 |
| Waist circumference, cm | 76.8 (8.7) | 79.1 (9.0) | 80.9 (9.5) | <0.001 | 76.9 (8.8) | 78.9 (9.1) | 80.9 (9.4) | <0.001 |
| Diabetes, % | 2.6 | 3.1 | 5.2 | <0.001 | 3.2 | 3.9 | 4.0 | 0.015 |
| Hypertension, % | 5.6 | 22.3 | 63.9 | <0.001 | 9.7 | 25.0 | 58.3 | <0.001 |
| Antihypertensive treatment, % | 0.8 | 3.4 | 15.4 | <0.001 | 1.1 | 4.8 | 15.8 | <0.001 |
| Family history of cardiovascular disease, % | 17.5 | 19.7 | 23.4 | <0.001 | 17.9 | 18.6 | 24.1 | <0.001 |

cumDBP, cumulative diastolic blood pressure; cumSBP, cumulative systolic blood pressure; MET-h/d, metabolic equivalent task-hour/day.

The 2004–2008 baseline characteristics are presented in the table. Data were presented as mean (standard deviation) or percentage, with adjustments for age, sex, and study areas.

**Table S2.** Associations between per 1-SD increment in blood pressure measures and incident intracerebral haemorrhage by age

|  | **Cases** | **Incidence rate (per 1000 person-years)** | **Model 1** | **Model 2** | **P value^*^** |
| --- | --- | --- | --- | --- | --- |
| **≥65 y** | 65 | 4.52 |  |  |  |
| **SBP** |  |  |  |  |  |
| Mean, per 19.5 mmHg |  |  | **1.55 (1.21, 1.98)** | 1.36 (0.85, 2.16) | 0.203 |
| MIN, per 17.8 mmHg |  |  | **1.37 (1.08, 1.74)** | 1.01 (0.70, 1.46) | 0.955 |
| MAX, per 23.3 mmHg |  |  | **1.65 (1.29, 2.12)** | **1.72 (1.09, 2.71)** | 0.020 |
| SD, per 6.8 mmHg |  |  | **1.38 (1.11, 1.71)** | 1.26 (0.98, 1.60) | 0.068 |
| CV, per 4.5% |  |  | **1.32 (1.06, 1.66)** | 1.25 (0.99, 1.59) | 0.062 |
| ARV, per 8.4 mmHg |  |  | **1.29 (1.05, 1.59)** | 1.16 (0.92, 1.46) | 0.203 |
| cumSBP, per 191.9 mmHg×year | |  | **1.80 (1.36, 2.37)** | **1.68 (1.14, 2.45)** | 0.008 |
| **DBP** |  |  |  |  |  |
| Mean, per 10.4 mmHg |  |  | **1.59 (1.23, 2.05)** | 1.24 (0.78, 1.97) | 0.371 |
| MIN, per 10.2 mmHg |  |  | **1.40 (1.08, 1.81)** | 0.96 (0.66, 1.39) | 0.823 |
| MAX, per 11.8 mmHg |  |  | **1.68 (1.30, 2.16)** | 1.56 (0.98, 2.48) | 0.061 |
| SD, per 3.6 mmHg |  |  | **1.33 (1.08, 1.65)** | 1.22 (0.96, 1.53) | 0.102 |
| CV, per 4.4% |  |  | 1.22 (0.99, 1.51) | 1.17 (0.94, 1.46) | 0.165 |
| ARV, per 4.6 mmHg |  |  | 1.19 (0.96, 1.49) | 1.07 (0.84, 1.36) | 0.594 |
| cumDBP, per 104.4 mmHg×year | |  | **1.80 (1.35, 2.41)** | **1.56 (1.05, 2.31)** | 0.027 |
| **<65 y** | 56 | 1.24 |  |  |  |
| **SBP** |  |  |  |  |  |
| Mean, per 19.5 mmHg |  |  | **2.07 (1.61, 2.66)** | 1.17 (0.67, 2.04) | 0.578 |
| MIN, per 17.8 mmHg |  |  | **1.79 (1.39, 2.30)** | 0.93 (0.63, 1.38) | 0.735 |
| MAX, per 23.3 mmHg |  |  | **2.15 (1.69, 2.74)** | 1.58 (0.87, 2.85) | 0.133 |
| SD, per 6.8 mmHg |  |  | **1.70 (1.37, 2.12)** | 1.21 (0.91, 1.61) | 0.198 |
| CV, per 4.5% |  |  | **1.53 (1.21, 1.95)** | 1.16 (0.89, 1.52) | 0.269 |
| ARV, per 8.4 mmHg |  |  | **1.63 (1.32, 2.01)** | 1.18 (0.91, 1.54) | 0.218 |
| cumSBP, per 191.9 mmHg×year | |  | **2.30 (1.77, 2.98)** | **1.69 (1.16, 2.46)** | 0.007 |
| **DBP** |  |  |  |  |  |
| Mean, per 10.4 mmHg |  |  | **2.04 (1.59, 2.61)** | 1.15 (0.68, 1.96) | 0.597 |
| MIN, per 10.2 mmHg |  |  | **1.89 (1.47, 2.44)** | 1.07 (0.73, 1.57) | 0.729 |
| MAX, per 11.8 mmHg |  |  | **2.11 (1.65, 2.71)** | 1.40 (0.78, 2.50) | 0.258 |
| SD, per 3.6 mmHg |  |  | **1.44 (1.15, 1.82)** | 1.07 (0.82, 1.39) | 0.630 |
| CV, per 4.4% |  |  | 1.25 (0.98, 1.60) | 1.05 (0.81, 1.37) | 0.691 |
| ARV, per 4.6 mmHg |  |  | **1.43 (1.14, 1.79)** | 1.10 (0.85, 1.42) | 0.450 |
| cumDBP, per 104.4 mmHg×year | |  | **2.19 (1.70, 2.82)** | **1.61 (1.12, 2.31)** | 0.010 |

ARV, average real variability; cumDBP, cumulative diastolic blood pressure; cumSBP, cumulative systolic blood pressure; CV, coefficient of variation; MAX, maximum; MIN, minimum; SD, standard deviation.

The hazard ratios and 95% confidence intervals are presented in the table, with the Cox model stratified by sex and study areas (10 groups) and the same covariates adjusted as shown in Table 2.

* This column displays the P values of model 2. The cut-off α value for Bonferroni correction = 0.05/14 = 0.004.

**Table S3.** Associations between per 1-SD increment in blood pressure measures and incident intracerebral haemorrhage by sex

|  | **Cases** | **Incidence rate (per 1000 person-years)** | **Model 1** | **Model 2** | **P value^*^** |
| --- | --- | --- | --- | --- | --- |
| **Men** | 61 | 2.72 |  |  |  |
| **SBP** |  |  |  |  |  |
| Mean, per 19.5 mmHg |  |  | **1.75 (1.34, 2.30)** | 1.27 (0.72, 2.24) | 0.400 |
| MIN, per 17.8 mmHg |  |  | **1.52 (1.15, 1.99)** | 0.93 (0.61, 1.42) | 0.748 |
| MAX, per 23.3 mmHg |  |  | **1.87 (1.42, 2.47)** | 1.73 (0.97, 3.10) | 0.065 |
| SD, per 6.8 mmHg |  |  | **1.56 (1.21, 2.01)** | 1.29 (0.96, 1.72) | 0.094 |
| CV, per 4.5% |  |  | **1.47 (1.13, 1.90)** | 1.28 (0.97, 1.69) | 0.076 |
| ARV, per 8.4 mmHg |  |  | **1.51 (1.20, 1.91)** | 1.26 (0.96, 1.65) | 0.091 |
| cumSBP, per 191.9 mmHg×year | |  | **2.06 (1.52, 2.78)** | **1.71 (1.14, 2.56)** | 0.010 |
| **DBP** |  |  |  |  |  |
| Mean, per 10.4 mmHg |  |  | **1.72 (1.33, 2.24)** | 1.50 (0.92, 2.45) | 0.107 |
| MIN, per 10.2 mmHg |  |  | **1.53 (1.17, 2.00)** | 1.06 (0.72, 1.58) | 0.753 |
| MAX, per 11.8 mmHg |  |  | **1.78 (1.38, 2.30)** | **1.91 (1.17, 3.12)** | 0.010 |
| SD, per 3.6 mmHg |  |  | **1.40 (1.11, 1.75)** | 1.26 (0.97, 1.62) | 0.080 |
| CV, per 4.4% |  |  | **1.29 (1.02, 1.63)** | 1.21 (0.94, 1.55) | 0.139 |
| ARV, per 4.6 mmHg |  |  | **1.43 (1.12, 1.83)** | 1.25 (0.96, 1.64) | 0.101 |
| cumDBP, per 104.4 mmHg×year | |  | **1.95 (1.46, 2.60)** | **1.82 (1.22, 2.71)** | **0.003** |
| **Women** | 60 | 1.61 |  |  |  |
| **SBP** |  |  |  |  |  |
| Mean, per 19.5 mmHg |  |  | **1.86 (1.43, 2.40)** | 1.15 (0.70, 1.90) | 0.578 |
| MIN, per 17.8 mmHg |  |  | **1.64 (1.27, 2.10)** | 1.00 (0.69, 1.44) | 1.000 |
| MAX, per 23.3 mmHg |  |  | **1.91 (1.48, 2.47)** | 1.33 (0.75, 2.34) | 0.331 |
| SD, per 6.8 mmHg |  |  | **1.41 (1.13, 1.74)** | 1.09 (0.83, 1.43) | 0.521 |
| CV, per 4.5% |  |  | **1.27 (1.01, 1.59)** | 1.06 (0.82, 1.38) | 0.639 |
| ARV, per 8.4 mmHg |  |  | **1.36 (1.09, 1.69)** | 1.08 (0.83, 1.41) | 0.562 |
| cumSBP, per 191.9 mmHg×year | |  | **2.08 (1.59, 2.72)** | **1.60 (1.11, 2.32)** | 0.012 |
| **DBP** |  |  |  |  |  |
| Mean, per 10.4 mmHg |  |  | **1.84 (1.42, 2.39)** | 0.90 (0.52, 1.56) | 0.699 |
| MIN, per 10.2 mmHg |  |  | **1.70 (1.30, 2.21)** | 0.89 (0.59, 1.34) | 0.581 |
| MAX, per 11.8 mmHg |  |  | **1.87 (1.45, 2.42)** | 0.97 (0.51, 1.85) | 0.928 |
| SD, per 3.6 mmHg |  |  | **1.32 (1.04, 1.67)** | 1.07 (0.82, 1.41) | 0.615 |
| CV, per 4.4% |  |  | 1.17 (0.92, 1.50) | 1.09 (0.83, 1.42) | 0.544 |
| ARV, per 4.6 mmHg |  |  | 1.27 (1.00, 1.59) | 1.08 (0.83, 1.41) | 0.574 |
| cumDBP, per 104.4 mmHg×year | |  | **2.03 (1.55, 2.65)** | 1.44 (0.98, 2.13) | 0.063 |

ARV, average real variability; cumDBP, cumulative diastolic blood pressure; cumSBP, cumulative systolic blood pressure; CV, coefficient of variation; MAX, maximum; MIN, minimum; SD, standard deviation.

The hazard ratios and 95% confidence intervals are presented in the table, with the Cox model stratified by age (5-year intervals) and study areas (10 groups) and the same covariates adjusted as shown in Table 2.

* This column displays the P values of model 2. The cut-off α value for Bonferroni correction = 0.05/14 = 0.004.

**Table S4.** Associations between per 1-SD increment in blood pressure measures and incident intracerebral haemorrhage by area of residence

|  | **Cases** | **Incidence rate (per 1000 person-years)** | **Model 1** | **Model 2** | **P value^*^** |
| --- | --- | --- | --- | --- | --- |
| **Rural** | 93 | 2.42 |  |  |  |
| **SBP** |  |  |  |  |  |
| Mean, per 19.5 mmHg |  |  | **1.77 (1.46, 2.15)** | 1.15 (0.75, 1.74) | 0.524 |
| MIN, per 17.8 mmHg |  |  | **1.56 (1.28, 1.90)** | 0.93 (0.69, 1.26) | 0.637 |
| MAX, per 23.3 mmHg |  |  | **1.87 (1.54, 2.27)** | **1.55 (1.02, 2.38)** | 0.041 |
| SD, per 6.8 mmHg |  |  | **1.49 (1.26, 1.77)** | 1.21 (0.99, 1.49) | 0.066 |
| CV, per 4.5% |  |  | **1.37 (1.14, 1.65)** | 1.19 (0.98, 1.46) | 0.080 |
| ARV, per 8.4 mmHg |  |  | **1.43 (1.22, 1.69)** | 1.18 (0.97, 1.43) | 0.098 |
| cumSBP, per 191.9 mmHg×year | |  | **2.06 (1.68, 2.53)** | **1.71 (1.28, 2.28)** | **<0.001** |
| **DBP** |  |  |  |  |  |
| Mean, per 10.4 mmHg |  |  | **1.79 (1.47, 2.18)** | 1.25 (0.83, 1.88) | 0.288 |
| MIN, per 10.2 mmHg |  |  | **1.68 (1.38, 2.06)** | 1.10 (0.81, 1.50) | 0.544 |
| MAX, per 11.8 mmHg |  |  | **1.79 (1.48, 2.18)** | 1.35 (0.86, 2.11) | 0.194 |
| SD, per 3.6 mmHg |  |  | **1.25 (1.04, 1.50)** | 1.04 (0.85, 1.28) | 0.683 |
| CV, per 4.4% |  |  | 1.11 (0.92, 1.34) | 1.03 (0.84, 1.25) | 0.774 |
| ARV, per 4.6 mmHg |  |  | **1.22 (1.01, 1.48)** | 1.04 (0.84, 1.28) | 0.740 |
| cumDBP, per 104.4 mmHg×year | |  | **2.03 (1.65, 2.50)** | **1.73 (1.29, 2.31)** | **<0.001** |
| **Urban** | 28 | 1.32 |  |  |  |
| **SBP** |  |  |  |  |  |
| Mean, per 19.5 mmHg |  |  | **1.74 (1.08, 2.83)** | 1.22 (0.55, 2.69) | 0.623 |
| MIN, per 17.8 mmHg |  |  | 1.52 (0.96, 2.40) | 1.02 (0.54, 1.91) | 0.950 |
| MAX, per 23.3 mmHg |  |  | **1.75 (1.07, 2.86)** | 1.18 (0.47, 2.96) | 0.724 |
| SD, per 6.8 mmHg |  |  | 1.31 (0.87, 1.97) | 1.08 (0.68, 1.70) | 0.745 |
| CV, per 4.5% |  |  | 1.24 (0.83, 1.87) | 1.08 (0.71, 1.66) | 0.710 |
| ARV, per 8.4 mmHg |  |  | 1.41 (0.93, 2.13) | 1.22 (0.77, 1.93) | 0.405 |
| cumSBP, per 191.9 mmHg×year | |  | 1.56 (0.95, 2.58) | 0.94 (0.44, 2.01) | 0.873 |
| **DBP** |  |  |  |  |  |
| Mean, per 10.4 mmHg |  |  | 1.53 (1.00, 2.35) | 0.92 (0.43, 1.96) | 0.824 |
| MIN, per 10.2 mmHg |  |  | 1.30 (0.84, 2.02) | 0.72 (0.39, 1.34) | 0.304 |
| MAX, per 11.8 mmHg |  |  | **1.71 (1.13, 2.57)** | 1.39 (0.68, 2.86) | 0.372 |
| SD, per 3.6 mmHg |  |  | **1.62 (1.12, 2.35)** | 1.42 (0.94, 2.15) | 0.092 |
| CV, per 4.4% |  |  | **1.55 (1.04, 2.32)** | 1.41 (0.93, 2.13) | 0.107 |
| ARV, per 4.6 mmHg |  |  | **1.57 (1.09, 2.27)** | 1.36 (0.91, 2.03) | 0.132 |
| cumDBP, per 104.4 mmHg×year | |  | 1.45 (0.92, 2.29) | 0.79 (0.37, 1.67) | 0.538 |

ARV, average real variability; cumDBP, cumulative diastolic blood pressure; cumSBP, cumulative systolic blood pressure; CV, coefficient of variation; MAX, maximum; MIN, minimum; SD, standard deviation.

The hazard ratios and 95% confidence intervals are presented in the table, with the Cox model stratified by age (5-year intervals), sex, and study areas (10 groups) and the same covariates adjusted as shown in Table 2.

* This column displays the P values of model 2. The cut-off α value for Bonferroni correction = 0.05/14 = 0.004.

**Table S5.** Associations between per 1-SD increment in blood pressure measures and incident intracerebral haemorrhage in participants without diabetes

|  | **Cases** | **Incidence rate (per 1000 person-years)** | **Model 1** | **Model 2** | **P value^*^** |
| --- | --- | --- | --- | --- | --- |
| **SBP** | 102 | 1.89 |  |  |  |
| Mean, per 19.5 mmHg |  |  | **2.01 (1.64, 2.46)** | **1.52 (1.02, 2.27)** | 0.037 |
| MIN, per 17.8 mmHg |  |  | **1.78 (1.46, 2.18)** | 1.14 (0.84, 1.55) | 0.411 |
| MAX, per 23.3 mmHg |  |  | **2.05 (1.68, 2.50)** | **1.79 (1.21, 2.65)** | 0.004 |
| SD, per 6.8 mmHg |  |  | **1.52 (1.28, 1.80)** | 1.17 (0.95, 1.46) | 0.146 |
| CV, per 4.5% |  |  | **1.39 (1.16, 1.67)** | 1.15 (0.93, 1.41) | 0.194 |
| ARV, per 8.4 mmHg |  |  | **1.40 (1.19, 1.65)** | 1.09 (0.89, 1.34) | 0.379 |
| cumSBP, per 191.9 mmHg×year | |  | **2.23 (1.82, 2.73)** | **1.86 (1.40, 2.47)** | **<0.001** |
| **DBP** | 102 | 1.89 |  |  |  |
| Mean, per 10.4 mmHg |  |  | **1.98 (1.63, 2.40)** | **1.70 (1.15, 2.53)** | 0.008 |
| MIN, per 10.2 mmHg |  |  | **1.87 (1.53, 2.29)** | 1.33 (0.97, 1.82) | 0.079 |
| MAX, per 11.8 mmHg |  |  | **1.93 (1.60, 2.34)** | **1.79 (1.22, 2.64)** | **0.003** |
| SD, per 3.6 mmHg |  |  | **1.30 (1.09, 1.55)** | 1.05 (0.86, 1.29) | 0.618 |
| CV, per 4.4% |  |  | 1.14 (0.95, 1.37) | 1.02 (0.83, 1.24) | 0.871 |
| ARV, per 4.6 mmHg |  |  | **1.25 (1.04, 1.50)** | 1.00 (0.81, 1.24) | 0.970 |
| cumDBP, per 104.4 mmHg×year | |  | **2.14 (1.76, 2.62)** | **1.89 (1.42, 2.51)** | **<0.001** |

ARV, average real variability; cumDBP, cumulative diastolic blood pressure; cumSBP, cumulative systolic blood pressure; CV, coefficient of variation; MAX, maximum; MIN, minimum; SD, standard deviation.

The hazard ratios and 95% confidence intervals are presented in the table, with the Cox model stratified by age (5-year intervals), sex, and study areas (10 groups). The same covariates were adjusted as shown in Table 2 except for the prevalence of diabetes.

* This column displays the P values of model 2. The cut-off α value for Bonferroni correction = 0.05/14 = 0.004.

**Table S6.** Sensitivity analysis of the associations between per 1-SD increment in blood pressure measures and incident intracerebral hemorrhage

|  | **Model 1** | **Model 2** | **P value^*^** |
| --- | --- | --- | --- |
| **SBP** |  |  |  |
| Mean, per 19.5 mmHg | **1.81 (1.52, 2.15)** | 1.25 (0.87, 1.79) | 0.234 |
| MIN, per 17.8 mmHg | **1.57 (1.32, 1.87)** | 0.96 (0.74, 1.25) | 0.760 |
| MAX, per 23.3 mmHg | **1.92 (1.61, 2.28)** | **1.66 (1.15, 2.39)** | 0.007 |
| SD, per 6.8 mmHg | **1.51 (1.30, 1.76)** | **1.23 (1.02, 1.48)** | 0.026 |
| CV, per 4.5% | **1.40 (1.19, 1.64)** | **1.20 (1.01, 1.43)** | 0.039 |
| ARV, per 8.4 mmHg | **1.45 (1.26, 1.68)** | **1.20 (1.01, 1.42)** | 0.040 |
| cumSBP, per 191.9 mmHg×year | **2.01 (1.68, 2.42)** | **1.62 (1.25, 2.10)** | **<0.001** |
| **DBP** |  |  |  |
| Mean, per 10.4 mmHg | **1.81 (1.52, 2.15)** | 1.26 (0.89, 1.79) | 0.192 |
| MIN, per 10.2 mmHg | **1.65 (1.38, 1.97)** | 1.04 (0.79, 1.35) | 0.792 |
| MAX, per 11.8 mmHg | **1.85 (1.56, 2.20)** | **1.54 (1.07, 2.19)** | 0.019 |
| SD, per 3.6 mmHg | **1.35 (1.16, 1.58)** | 1.14 (0.96, 1.36) | 0.136 |
| CV, per 4.4% | **1.21 (1.03, 1.42)** | 1.11 (0.94, 1.32) | 0.217 |
| ARV, per 4.6 mmHg | **1.31 (1.12, 1.54)** | 1.11 (0.93, 1.33) | 0.245 |
| cumDBP, per 104.4 mmHg×year | **1.98 (1.65, 2.37)** | **1.59 (1.23, 2.07)** | **<0.001** |

ARV, average real variability; cumDBP, cumulative diastolic blood pressure; cumSBP, cumulative systolic blood pressure; CV, coefficient of variation; MAX, maximum; MIN, minimum; SD, standard deviation.

The hazard ratios and 95% confidence intervals are presented in the table, with the Cox model stratified by age (5-year intervals), sex, and study areas (10 groups). Both model 1 and model 2 were additionally adjusted for **heart rate variability** (SD), and other covariates were the same as shown in Table 2.

* This column displays the P values of model 2. The cut-off α value for Bonferroni correction = 0.05/14 = 0.004.

**Table S7.** Sensitivity analysis of associations between per 1-SD increment in blood pressure measures and incident intracerebral haemorrhage for women

|  | **Model 1** | **Model 2** | **P value^*^** |
| --- | --- | --- | --- |
| **SBP** |  |  |  |
| Mean, per 19.5 mmHg | **1.85 (1.43, 2.40)** | 1.15 (0.70, 1.90) | 0.578 |
| MIN, per 17.8 mmHg | **1.63 (1.27, 2.10)** | 1.00 (0.69, 1.44) | 0.996 |
| MAX, per 23.3 mmHg | **1.91 (1.48, 2.46)** | 1.33 (0.75, 2.35) | 0.323 |
| SD, per 6.8 mmHg | **1.41 (1.14, 1.74)** | 1.09 (0.84, 1.43) | 0.510 |
| CV, per 4.5% | **1.27 (1.02, 1.60)** | 1.07 (0.82, 1.38) | 0.624 |
| ARV, per 8.4 mmHg | **1.36 (1.09, 1.68)** | 1.08 (0.83, 1.41) | 0.555 |
| cumSBP, per 191.9 mmHg×year | **2.08 (1.59, 2.72)** | **1.61 (1.11, 2.33)** | 0.012 |
| **DBP** |  |  |  |
| Mean, per 10.4 mmHg | **1.84 (1.42, 2.39)** | 0.89 (0.51, 1.56) | 0.691 |
| MIN, per 10.2 mmHg | **1.70 (1.30, 2.22)** | 0.89 (0.59, 1.34) | 0.575 |
| MAX, per 11.8 mmHg | **1.87 (1.45, 2.42)** | 0.97 (0.51, 1.84) | 0.928 |
| SD, per 3.6 mmHg | **1.32 (1.04, 1.67)** | 1.07 (0.82, 1.41) | 0.611 |
| CV, per 4.4% | 1.18 (0.92, 1.50) | 1.09 (0.83, 1.42) | 0.540 |
| ARV, per 4.6 mmHg | 1.27 (1.00, 1.59) | 1.08 (0.83, 1.41) | 0.579 |
| cumDBP, per 104.4 mmHg×year | **2.03 (1.55, 2.66)** | 1.45 (0.98, 2.13) | 0.062 |

ARV, average real variability; cumDBP, cumulative diastolic blood pressure; cumSBP, cumulative systolic blood pressure; CV, coefficient of variation; MAX, maximum; MIN, minimum; SD, standard deviation.

The hazard ratios and 95% confidence intervals are presented in the table, with the Cox model stratified by age (5-year intervals) and study areas (10 groups). Both model 1 and model 2 were additionally adjusted for **menopausal status** at 2013–2014 resurvey, and other covariates were the same as shown in Table 2.

* This column displays the P values of model 2. The cut-off α value for Bonferroni correction = 0.05/14 = 0.004.

**Table S8.** Sensitivity analysis of the incremental predictive performance of long-term BP measures for 5-year intracerebral hemorrhage risk

|  | Harrell's C | △Harrell's C | cNRI | rIDI (%) |
| --- | --- | --- | --- | --- |
| Basic model | 0.820 (0.778, 0.861) | — | — | — |
| Basic model+CV | 0.821 (0.779, 0.862) | 0.001 (-0.003, 0.004) | -0.038 (-0.234, 0.158) | 5.0 (-1.4, 11.4) |
| Basic model+ARV | 0.821 (0.779, 0.863) | 0.001 (-0.003, 0.005) | 0.025 (-0.166, 0.215) | 1.9 (-3.0, 6.9) |
| Basic model+Mean+SD+MIN+MAX | 0.823 (0.782, 0.864) | 0.003 (-0.004, 0.010) | 0.134 (-0.070, 0.337) | 11.8 (-2.6, 26.3) |
| Basic model+cumBP | 0.829 (0.790, 0.867) | 0.009 (-0.001, 0.019) | **0.288 (0.089, 0.486)** | **18.4 (6.3, 30.6)** |

ARV, average real variability; cNRI, continuous net reclassification improvement; cumBP, cumulative blood pressure; CV, coefficient of variation; MAX, maximum; MIN, minimum; rIDI, relative integrated discrimination improvement; SD, standard deviation.

The other statistical methods were the same as those displayed in Figure 1 and Table 3, with the addition of **heart rate variability** (SD) to the basic model.

**Table S9.** Sensitivity analysis of the incremental predictive performance of long-term BP measures for 5-year intracerebral hemorrhage risk for women

|  | Harrell's C | △Harrell's C | cNRI | rIDI (%) |
| --- | --- | --- | --- | --- |
| Basic model | 0.831 (0.774, 0.888) | — | — | — |
| Basic model+CV | 0.831 (0.774, 0.888) | 0.000 (-0.001, 0.001) | -0.121 (-0.388, 0.146) | 0.8 (-0.2, 1.9) |
| Basic model+ARV | 0.830 (0.775, 0.886) | -0.001 (-0.004, 0.003) | 0.081 (-0.212, 0.374) | -0.2 (-2.5, 2.2) |
| Basic model+Mean+SD+MIN+MAX | 0.829 (0.771, 0.887) | -0.002 (-0.013, 0.009) | 0.037 (-0.202, 0.275) | **19.9 (3.2, 36.7)** |
| Basic model+cumBP | 0.837 (0.785, 0.890) | 0.006 (-0.003, 0.016) | 0.181 (-0.074, 0.436) | **18.0 (1.9, 34.1)** |

ARV, average real variability; cNRI, continuous net reclassification improvement; cumBP, cumulative blood pressure; CV, coefficient of variation; MAX, maximum; MIN, minimum; rIDI, relative integrated discrimination improvement; SD, standard deviation.

The other statistical methods were the same as those displayed in Figure 1 and Table 3, with the addition of **menopausal status** at 2013–2014 resurvey to the basic model.
